# A Case of Oropharyngeal Carcinoma Accompanying a Presacral Malignant Epidermoid Cyst

**DOI:** 10.7759/cureus.69841

**Published:** 2024-09-21

**Authors:** Daiki Kitano, Yuki Komatsu, Makoto Omori

**Affiliations:** 1 Plastic Surgery, Yodogawa Christian Hospital, Osaka, JPN

**Keywords:** adjuvant chemoradiation therapy, cancer metastasis, head and neck neoplasms, presacral tumor, ­reconstructive surgery

## Abstract

Presacral epidermoid cysts are rare benign tumors that can undergo malignant transformation. Here, we report a case of squamous cell carcinoma (SCC) arising from a presacral epidermoid cyst that metastasized to the mesopharynx. A 66-year-old female presented with abdominal pain and fever, leading to the discovery of a 5 cm presacral epidermoid cyst. Since the tumor had invaded into the rectum and uterus, total pelvic exenteration followed by ileostomy was performed. Histopathological examination revealed poorly differentiated SCC, suggesting malignant transformation from the epithelial component of the epidermoid cyst. Four years after adjuvant radiotherapy (45Gy/18Fr), a 5 cm left cervical lymphadenopathy prompted the diagnosis of human papillomavirus (HPV)-negative mesopharyngeal carcinoma (lateral wall, T2N3b). Following neoadjuvant chemotherapy, the patient underwent tumor resection, neck dissection, and free radial forearm flap reconstruction. Histopathological analysis of the mesopharyngeal tumor and cervical lymph node revealed poorly differentiated SCC with cystic formation, resembling an epidermoid cyst, suggesting distant metastasis from the presacral malignant epidermoid cyst. Postoperative treatment included chemotherapy (FOLFOX (5-FU, leucovorin, oxaliplatin)) and radiotherapy (50Gy/25Fr). At one year and five months postoperatively, there has been no recurrence of the malignant tumor. Metastatic mesopharyngeal carcinoma originating from other organs is extremely rare. While there is no established adjuvant chemotherapy regimen for presacral malignant epidermoid cysts, we preferred FOLFOX as used in the treatment of unresectable colorectal cancer with distant metastases.

## Introduction

Epidermoid cysts (ECs), which typically develop on exposed areas of the skin, such as the trunk and face, are among the most common benign tumors encountered in daily clinical practice. As ECs consist of an epithelial wall, they have the potential to occur in any part of the body where epithelial tissue is present [1].

The treatment for ECs in exposed areas generally involves marginal resection along the epithelial wall. However, since ECs are benign, not all cases necessitate resection. Observation may be an option, but in some cases, the contents of the EC may become infected during this period, requiring emergency treatment. Infected ECs present with symptoms such as swelling, warmth, and pain, and may rupture, necessitating surgical drainage of purulent discharge.

Another reason for recommending the surgical removal of ECs is the potential for malignant transformation. Veenstra et al. reported three cases (0.16%) of squamous cell carcinoma (SCC) among 1,904 cases of ECs [2]. Although malignant transformation of EC is rare, there are cases where patients initially diagnosed with benign tumors, when monitored over an extended period, later present with distant metastasis and life-threatening conditions.

The presacral area, located between the sacrum and rectum, is a common site for tumors originating from embryonic tissues [3]. Among these tumors, those composed of epithelial components are termed presacral ECs. Like ECs in exposed areas, presacral ECs are benign in most cases, but there is ongoing debate regarding the necessity of treatment. Given their benign nature, many surgeons question whether it is justifiable to perform an invasive procedure to remove them.

While presacral ECs are typically benign, several cases of malignant transformation have been reported [4-6]. Due to the rarity of malignant transformation and limited number of cases, treatment strategies, including the extent of resection and the use of adjuvant chemoradiotherapy, are not well established. In this report, we report a case of oropharyngeal carcinoma arising from a presacral malignant EC and discuss the treatment strategy.

## Case presentation

**Figure 1:**
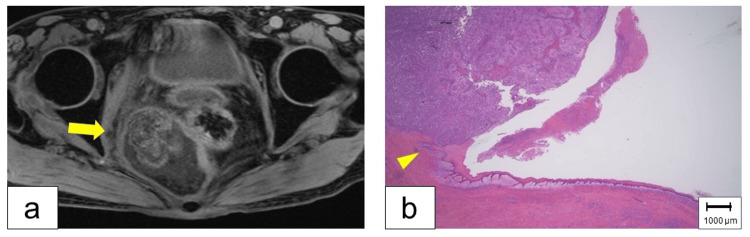
Presacral Epidermoid Cyst (a) Magnetic resonance imaging (T1-weighted) of the pelvis demonstrated a 5 cm tumor with heterogeneous signal intensity in the presacral space (arrow). (b) Histopathological examination of the excised tumor revealed squamous cell carcinoma. The arrowhead indicated the boundary between the intact epithelial layer and the malignant tumor.

A 66-year-old female presented to the emergency department with complaints of abdominal pain and fever. She had a history of alcohol consumption of three cups of shochu (Japanese distilled liquor) per day for 40 years and smoking one pack of cigarettes per day for 40 years. Her family history is notable for her mother’s death from breast cancer. Abdominal computed tomography (CT) and magnetic resonance imaging (MRI) revealed an irregularly shaped, 5 cm mass in the presacral area (Figure 1A). The tumor was infected and ruptured, leading to peritonitis.

Under general anesthesia, total pelvic exenteration following ileostomy was performed due to the invasion of tumor into the rectus and uterus. The pelvic cavity after exenteration was filled with an omental flap, resulting in the suppression of the infection. Pathological examination of the excised tumor revealed an EC with poorly differentiated SCC (Figure 1B). Adjuvant chemotherapy was proposed; however, the patient refused it. Six months postoperatively, the patient agreed to receive radiotherapy (45Gy/18Fr) due to recurrence of the tumor. Follow-up CT showed significant reduction in tumor size, but no complete remission.

**Figure 2:**
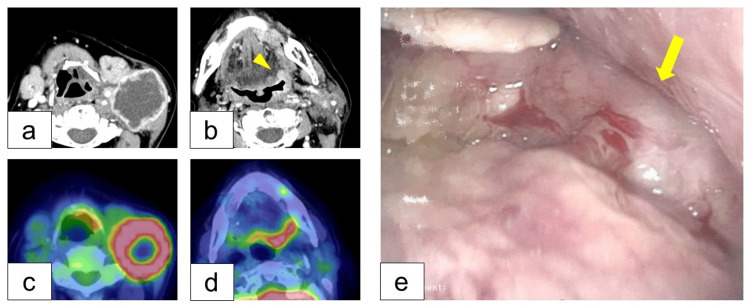
Cervical Lymph Node Metastasis and Mesopharyngeal Tumor Contrast-enhanced computed tomography (CT) showed a 5 cm tumor with ring enhancement in the left cervical lymph node (a) and the left oropharynx (b), both exhibiting increased fluorodeoxyglucose (FDG) uptake on positron emission tomography CT (c, d). (e) Pharyngoscopy revealed a hypervascular lesion with whitish exudate on the left wall of the oropharynx (arrow).

Four years after the initial surgery, the patient noticed a rapidly growing mass in her left neck. Contrast-enhanced CT revealed a 5 cm tumor with ring enhancement (Figure 2A) in the neck and a high-density area in the oropharynx (Figure 2B). Positron emission tomography (PET)-CT showed increased uptake of fluorodeoxyglucose (FDG) at the neck tumor (Figure 2C) and meshopharynx (Figure 2D). Pharyngoscopy revealed a 3 cm whitish lesion on the left lateral wall of oropharynx (Figure 2E). Biopsy of the neck tumor and oropharyngeal lesion confirmed poorly differentiated SCC (p16 negative). Further study found multiple cancers, including hypopharyngeal cancer (right pyriform sinus, cT2, SCC), esophageal cancer (the upper and middle thoracic esophagus, cT1a, SCC), and early gastric cancer (anterior wall, cTis, adenocarcinoma).

**Figure 3:**
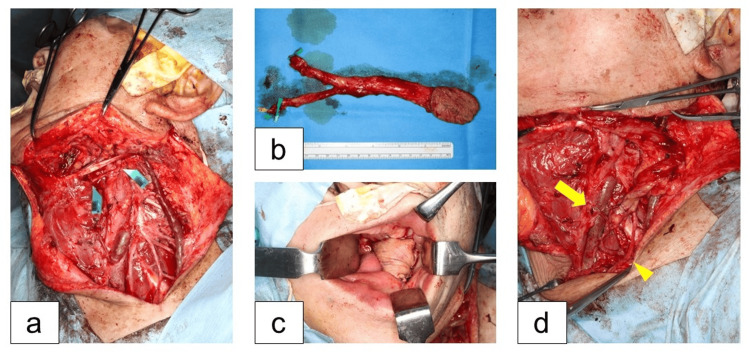
Operative Findings (a) Resected oropharyngeal tumor and cervical lymph node with hypoglossal nerve. Nerve gap reconstructed with an interpositional nerve graft. (b) Harvested 5 × 6 cm free radial forearm flap. (c) Reconstruction of the oropharyngeal defect with the flap. (d) Recipient vessels for the flap were the transverse cervical artery (arrowhead) and internal jugular vein (arrow).

The clinical diagnosis was human papillomavirus (HPV)-negative oropharyngeal cancer with cervical lymph node metastasis (left lateral wall, cT2N3b, SCC). After neoadjuvant chemotherapy with paclitaxel, carboplatin and cetuximab (PCE), the patient underwent tumor resection including hypoglossal nerve, neck dissection (Levels I-V), and free forearm flap transfer and nerve reconstruction (Figure 3).

**Figure 4:**
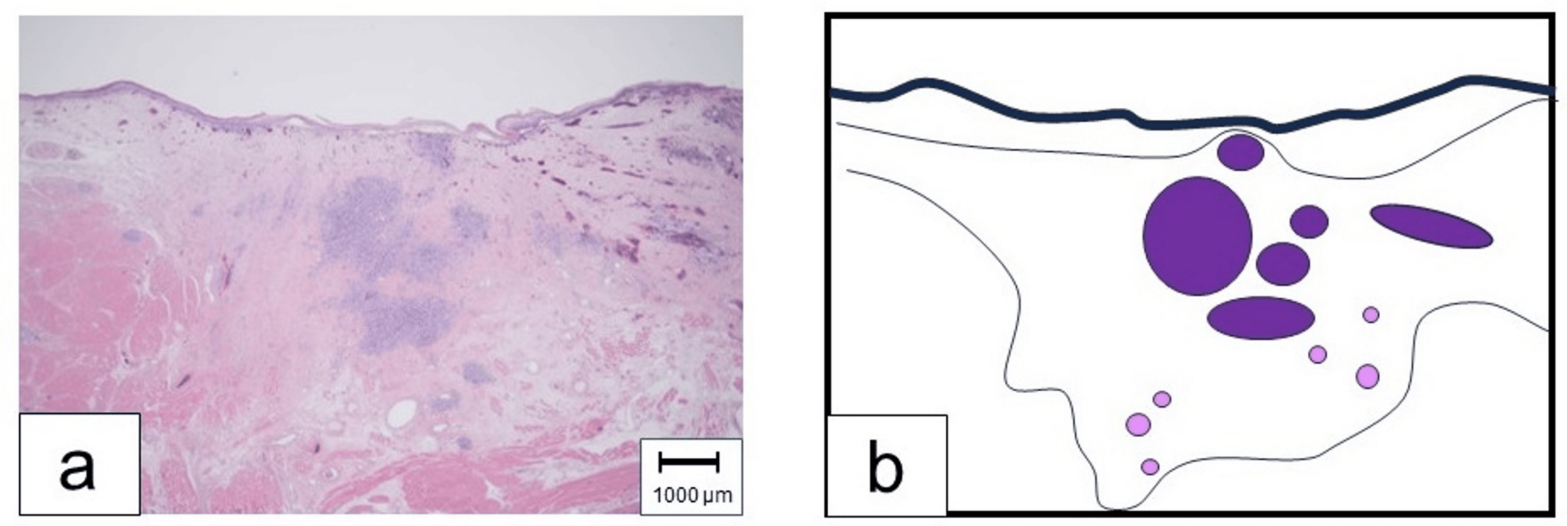
Histopathological Findings of the Mesopharyngeal Tumor Hematoxylin-Eosin stain (a) and schema (b) of the resected specimen. The lesion (purple circle, in schema) located beneath the mucosal epithelium of oropharynx. No continuity was evident between the lesion and the intact epithelium layer. The tumor formed cystic lesions resembling epidermoid cysts (pink circles, in schema).

Pathological examination of the excised lesion from the left lateral wall of the oropharynx revealed poorly differentiated SCC with cystic formation resembling an EC (Figure 4) along with extranodal extension of the tumor in the enlarged lymph node (pN3b). Since the lesion was located beneath the intact oropharyngeal epithelium layer, no continuity was observed. Pathologists suggested the possibility of distant metastasis from the presacral malignant EC.

For the treatment of other cancers, endoscopic submucosal dissection was performed for the gastric cancer and radiotherapy for hypopharyngeal and esophageal cancers. Considering the possibility of mesopharyngeal SCC originating from the presacral malignant EC, adjuvant chemoradiotherapy was necessary. We adopted FOLFOX (5-FU, leucovorin, oxaliplatin) which is indicated for the treatment of metastatic colorectal cancer. The radiation field was set from the neck to thorax, including the oro- and hypopharynx, cervical lymph nodes, and esophagus. Since the patient developed febrile neutropenia and grade 3 oral mucositis associated with chemoradiotherapy, the decision was made to discontinue adjuvant therapy at 50Gy/25Fr. One year and five months after the final surgery, no recurrence has been confirmed for any of the lesions. Although the patient has slight difficulty with her speech, she can eat normal meals and maintain her pre-surgery lifestyle.

**Figure 5:**
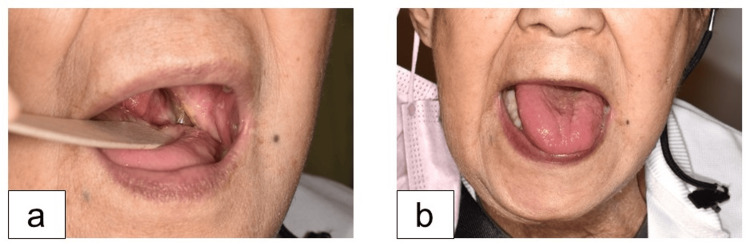
Postoperative One Year and Five Months (a) Free radial forearm flap transplanted to the lateral wall of oropharynx. (b) The tongue showed slight atrophy, but maintained mobility.

## Discussion

Pharyngeal cancer is associated with a history of alcohol and cigarette consumption [7]. Indeed, our patient was a heavy drinker and smoker. Although she was diagnosed with HPV-negative oropharyngeal cancer, there has been an increasing incidence of HPV-positive in literature. Sometimes, HPV-positive oropharyngeal cancer patients present as a cervical lymph node metastatic cancer with unknown origin [8,9]. Therefore, once cervical lymph node metastasis is identified, we should seek the possibility of oropharyngeal cancer [10].

While metastatic oropharyngeal cancer is extremely rare, there are several reported cases. Shin et al. conducted a single-center cohort study of 1,445 patients with head and neck cancer [11]. Among 29 cases of metastatic cancers, six cases were metastatic oropharyngeal cancers. Pathological examination of six cases revealed that the origin of metastatic oropharyngeal cancers was lung in three cases, and liver, stomach, breast in one case, respectively. However, as the present study was based on pathological specimens, clinical descriptions are limited. To our knowledge, there are two reported cases of metastatic oropharyngeal cancers [12,13]. The origin of tumors were colorectal adenocarcinoma and hepatocellular carcinoma, respectively.

In our case, all the pathological diagnosis of oropharyngeal cancer, cervical lymph node, and presacral malignant EC was poorly differentiated SCC. Although the pathological findings were consistent with the metastatic cancer, it was impractical to conclude the distant metastasis from presacral malignant EC. Since the majority of primary oropharyngeal cancer was diagnosed as SCC, we could not deny the possibility of primary oropharngeal cancer. However, considering the pathological findings (cystic lesions resembling ECs were present in the oropharynx, and the absence of continuity between the lesion and the normal nasopharyngeal epithelium), we supported the idea that the most probable origin of oropharyngeal SCC was the presacral malignant EC.

Since extranodular extension of cervical lymph node metastasis was observed, postoperative chemoradiotherapy was performed. Weekly cisplatin-based chemotherapy is commonly used for head and neck cancer [14], however, there is no established chemotherapy regimen for presacral malignant ECs. We adopted FOLFOX, which is indicated for unresectable colorectal cancer with distant metastases, and for esophageal cancer [15]. Compared to cisplatin, oxaliplatin does not require large-volume intravenous fluid infusion or hospitalization. In our case, FOLFOX was administered along with radiotherapy at 50Gy/25Fr, until febrile neutropenia and grade 3 oral mucositis occurred. At one year and five months postoperatively, there has been no recurrence of the cancer.

## Conclusions

We present a case of metastatic oropharyngeal cancer originating from a presacral malignant epidermoid cyst. The ruptured presacral malignant epidermoid cyst, which had disseminated the malignant cells in the abdominal cavity, led to hematogenous metastasis to the oropharynx, followed by metastasis to the cervical lymph nodes via lymphatic flow. After resection of the oropharyngeal tumor, neck dissection, and free radial forearm flap reconstruction, we performed chemoradiotherapy using FOLFOX, which is indicated for unresectable colorectal cancer with distant metastases. At one year and five months postoperatively, there has been no recurrence.
